# Biomaterial-Assisted 3D In Vitro Tumor Models: From Organoid towards Cancer Tissue Engineering Approaches

**DOI:** 10.3390/cancers15041201

**Published:** 2023-02-14

**Authors:** Nicola Contessi Negrini, Alessandro Franchi, Serena Danti

**Affiliations:** 1 Department of Bioengineering, Imperial College London, London SW7 2BX, UK; 2 Department of Translational Research and of New Technologies in Medicine and Surgery, University of Pisa, 56126 Pisa, Italy; 3 Department of Civil and Industrial Engineering, University of Pisa, 56126 Pisa, Italy

Cancers are a leading cause of death around the world, accounting for nearly 10 million deaths yearly. Scientists across the globe are intensifying more and more of their efforts to understand tumor development, to develop early-stage diagnostic (and theragnostic) methods, as well as to identify new and personalized biomarkers to obtain novel and more efficient drugs. The connecting link of all these aspects is the availability of reliable, reproducible, cost-effective, and ethically sustainable models. Currently used in vivo animal models and in vitro bi-dimensional (2D) models are affected by drawbacks that are hindering the understanding of tumor structural and biological complexity and the development of efficient treatments. In recent years, biomaterial-assisted three-dimensional (3D) in vitro modelling has gained tremendous momentum as a more reliable and biomimetic alternative to better understand tumor biology and develop new therapeutics or screen drugs and drug combinations for personalized medicine approaches. The growing knowledge produced in the field of tissue engineering has lately opened the way for cancer tissue engineering [1]. The multi- and inter-disciplinary nature of this topic needs to fill a methodology gap, which involves bringing the cancer biology community closer to the field of biomaterials science and *vice versa*. This is the objective of our Special Issue, in which we collected a series of 13 papers (7 research papers, 4 reviews, 1 systematic review, and 1 commentary) published by international leaders in relation to the development and application of 3D in vitro tumor models using biomaterials as instructive elements.

Furthermore, 3D biomaterial-assisted in vitro models can be used to culture multiple cell populations using biomaterials to recapitulate the 3D structure of the tissue in vivo, not achievable by culturing cells on traditional 2D plastic substrates. Several biomaterials or biomaterial combinations can be used to culture different cells to in vitro mimic the complexity of specific cancers, replicating cell–cell interactions, cell–extracellular matrix (ECM) interactions, and vascularization, which is not largely possible using traditional 2D culture plastics as substrates [2,3]. The different structural and biological complexity obtained with 3D in vitro models not only emulates the in vivo morphology, but it also increases the functional similarity of the pathophysiological in vivo tissue/organ. For instance, the drug sensitivity of tissues in vivo, such as the response to innovative immunotherapy, is better mimicked and replicated by 3D in vitro models compared to 2D in vitro models [4].

Betriu et al. [5] studied the internalization and degradation of epithelial growth factor receptors (EGFRs) of pancreatic ductal adenocarcinoma after treatment with Erlotinib and compared the response of cells cultured on 2D plastics and 3D self-assembling peptide scaffolds. The authors demonstrated how Erlotinib treatment promoted epidermal growth factor (EGFR) degradation by cells cultured in 3D cultures, but not on 2D cultures, which proves how the 3D environment allowed for an improved mimicking of in vivo cellular morphology, matrix dimensionality ad stiffness, molecular gradient, and response to therapeutics. In order to prepare 3D in vitro models of soft and hard cancers, Tomar et al. [6] used polyhydroxyalkanoates synthesized by microorganisms to fabricate 3D porous scaffolds via particulate leaching. The authors cultured breast and colon cancer cells and demonstrated a nutrient diffusion across the scaffolds and cell penetration in the biomaterial 3D structure, proving the suitability of these scaffolds as 3D biomaterial-assisted in vitro cancer models as more biomimetic alternatives to conventional cell cultures. The use of these in vitro models is particularly promising when patient-derived cancer cells are cultured in biomaterial scaffolds and used to develop a patient-specific 3D in vitro model, enabling the possibility of developing personalized and patient-specific cancer treatments [7]. However, the use of 3D models imposes new technical challenges that still need to be fully addressed; namely, biomaterial-assisted in vitro models that use highly reproducible, cost-effective, and adequate technologies for analysis are yet to be optimized. In this context, for example, Shembrey et al. developed a method to monitor intra-tumoral cell heterogeneity using the optical barcoding of patient-derived cancer cells in a 3D in vitro model of colorectal cancer [8].

Biomaterial-assisted 3D in vitro models of cancers have the great potential of overcoming the limitations of the currently used 2D in vitro model. However, developing biomaterial-assisted 3D models comes with several challenges that need to be carefully tackled to achieve an efficient model. Design parameters that need to be addressed include morphological and topographical cues, structural and mechanical cues, and chemical/biological cues to correctly mimic the in vivo tumor microenvironment found [9]. Wieland et al. prepared 3D aligned microfiber scaffolds via melt electrowriting to mimic brain structures and demonstrated the role of topography in determining cell behavior in a metastatic brain model [10]. The authors showed the importance of aligned microfibers to guide the formation of an in vitro relevant biomimetic model and applied the model to validate specific genotypes and their involvement in controlling cell morphology, durotaxis, adhesion, plasticity, and migration in a brain metastasis in vitro model. Mechanical cues are also fundamental to obtaining a relevant biomaterial-assisted in vitro cancer model for both soft and hard tissues, as in the case of bone metastasis and bone cancer [11]. For instance, we developed an in vitro osteosarcoma model using 3D-printed polyurethane and we tuned the porosity and mechanical properties of the scaffolds to achieve an optimal scaffold to promote cell colonization in the scaffold pores and osteogenic differentiation, which could be a platform for osteosarcoma study [12]. After optimizing the physico-mechanical properties of the scaffolds, we introduced biomimetic cues by pre-generating a bone-like ECM on the prepared scaffolds with osteo-induced mesenchymal stem cells [13]. The presence of biomimetic chemical/biological cues is indeed critical for an efficient biomaterial-assisted 3D in vitro model. In the context of the 3D in vitro model of ovarian cell cancer [14], Gupta et al. developed and compared different polymeric scaffolds and highlighted the importance of mimicking ECM components in vitro to accurately mimic the in vivo tumor [15]. Finally, Wishart et al. demonstrated the importance of a fibronectin coating on their polyurethane scaffolds to develop an in vitro model capable of recapitulating hypoxic conditions in 3D pancreatic cancer models to study the effects of radiotherapy treatment [16].

Biomaterial-assisted in vitro models have the great potential of allowing the biological and structural complexity of tumors to be recapitulated in vitro, thus opening new frontiers never investigated before with traditionally used 2D in vitro models and in vivo animal models. The continuous innovation in biomaterials science and new biomolecular tools for the characterization of in vitro models will critically contribute to improving our understanding of cancer and develop new therapeutics to treat such dismal conditions. We hope that this Special Issue will motivate cancer biology scientists and biomaterials and tissue engineers to join forces to tackle this challenging research field, in order to make advances in cancer understanding and treatment.
